# Human-Biting *Ixodes* Ticks and Pathogen Prevalence from California, Oregon, and Washington

**DOI:** 10.1089/vbz.2018.2323

**Published:** 2019-01-28

**Authors:** Guang Xu, Patrick Pearson, Elizabeth Dykstra, Elizabeth S. Andrews, Stephen M. Rich

**Affiliations:** ^1^ Laboratory of Medical Zoology, Department of Microbiology, University of Massachusetts–Amherst, Amherst, Massachusetts.; ^2^ Zoonotic Disease Program, Washington State Department of Health, Olympia, Washington.; ^3^ Vector-Borne Disease Section, California Department of Public Health, Elk Grove, California.

**Keywords:** human-biting *Ixodes*, pathogen, California, Oregon, Washington

## Abstract

From July 2006 through August 2017, a passive surveillance study of *Ixodes* ticks submitted from California, Oregon, and Washington was conducted by the TickReport program at the University of Massachusetts, Amherst. In total, 549 human-biting *Ixodes* ticks were submitted comprising both endemic and nonendemic species. We found that 430 endemic ticks were from 3 *Ixodes* species: *Ixodes pacificus*, *Ixodes spinipalpis*, and *Ixodes angustus*, whereas *Ixodes scapularis* (*n* = 111) was the most common species among the 119 nonendemic ticks. The submission peak for nymphal *I. pacificus* and *I. spinipalpis* was June, while submission peak for adult *I. pacificus* and nymphal *I. angustus* was April and September, respectively. Endemic ticks commonly attached to the lower extremities of their victims, and individuals younger than 9 years old were frequently bitten. The infection prevalence of *Borrelia burgdorferi sensu lato*, *Borrelia miyamotoi*, and *Anaplasma phagocytophilum* in *I. pacificus* ticks was 1.31%, 1.05%, and 0.52%, respectively, and the prevalence of *B. burgdorferi s. l.* and *A. phagocytophilum* in *I. spinipalpis* ticks was 14.29% and 10.71%, respectively. Furthermore, two species within the *B. burgdorferi s. l.* complex were detected in West Coast ticks: *B. burgdorferi sensu stricto* and *Borrelia lanei. I. spinipalpis* had the highest *Borrelia* prevalence among endemic ticks, and it was caused exclusively by *B. lanei*. *Borrelia mayonii*, *Babesia microti*, and *Ehrlichia muris*-like agent were not detected in these endemic ticks. In this study, we show that many nonendemic *Ixodes* ticks (119/549) are most likely acquired from travel to a different geographic region. We report cases of conventionally recognized nonhuman feeders (*I. spinipalpis* and *I. angustus*) parasitizing humans. The highest pathogen prevalence in *I. spinipalpis* may indicate a larger public health threat than previously thought, and the enzootic life cycle and pathogenicity of *B. lanei* warrant further study.

## Introduction

The single prostriate genus *Ixodes* comprises medically important ticks throughout the world (Keirans and Clifford 1978, Piesman and Eisen 2008). Several members within the *Ixodes ricinus* complex are the primary tick vectors that are involved in transmission of human pathogens (Xu et al. 2003). For example, in the eastern and north-central United States, *Ixodes scapularis* is the primary vector of several pathogens, including *Borrelia burgdorferi* (Lyme disease), *Borrelia miyamotoi*, *Anaplasma phagocytophilum*, and *Babesia microti* (Piesman and Eisen 2008). In the western United States, *Ixodes pacificus* is the primary vector of these pathogens (Burgdorfer et al. 1985, Lane et al. 1994, Eisen et al. 2016).

The prevalence and ecology of Lyme disease in the western United States differs significantly from that in the northeastern United States. The overall incidence of Lyme disease in the western United States is 0.2 cases compared to 30–80 cases (per 100,000 persons per year) in the northeastern United States (Schwartz et al. 2017). There is also greater vector and spirochete diversity in the western United States than in the northeastern United States (Brown and Lane 1992). In the Northeast, Lyme spirochetes are primarily maintained in white-footed mice (*Peromyscus leucopus*) and other small mammals by *I. scapularis* (Spielman et al. 1985). However, in the western United States, *Peromyscus* mice have low tick load and low prevalence of *B. burgdorferi sensu lato* (*s. l.*) (Brown and Lane 1992), whereas, the western gray squirrel (*Sciurus griseus*) and dusky-footed woodrat (*Neotoma fuscipes*) appear to be the predominant reservoir hosts of *B. burgdorferi* (Salkeld et al. 2008, 2014a, Salkeld and Lane 2010). *I. pacificus* mainly serves as a “bridge” vector of spirochetes to humans (Burgdorfer et al. 1985, Lane and Lavoie 1988, Clover and Lane 1995). In California, *Borrelia* species are also maintained among dusky-footed woodrats, California kangaroo rats (*Dipodomys californicus*), and other small mammals by *Ixodes spinipalpis*, *Ixodes angustus*, and *Ixodes jellisoni*, although they rarely bite humans (Burgdorfer et al. 1985, Brown and Lane 1992, Lane et al. 1994).

In addition to differences in hosts and tick vectors, the species and population of Lyme spirochetes are more diverse in California (Girard et al. 2009). Up to now, only four *Borrelia* species within *B. burgdorferi s. l.* have been found in the northeastern and upper midwestern United States: *Borrelia andersonii*, *B. burgdorferi sensu stricto* (*s. s.*), *Borrelia kurtenbachii*, and *Borrelia mayonii*, with *B. burgdorferi s. s.* representing the dominant species (Margos et al. 2010, 2014). Conversely, in addition to *B. burgdorferi s. s.*, several species within the *B. burgdorferi s. l.* complex, including *Borrelia americana* (Rudenko et al. 2009), *Borrelia bissettiae* (Postic et al. 1998), *Borrelia californensis* (Postic et al. 2007), and *Borrelia lanei* (Margos et al. 2017) have been found in the western United States. *B. burgdorferi s. s.* is the only species associated with Lyme borreliosis: it contains two genetically distinct, but phylogenetically related populations in the Northeast and Midwest (Qiu et al. 2002, Hoen et al. 2009). However, the population structure of California strains of *B. burgdorferi s. s.* is more heterogeneous than the Northeast strains (Girard et al. 2009).

Several tick surveillance studies have been conducted to connect tick data with the risk of tick-borne disease in the western United States, including research by traditional tick flagging (Lane et al. 2004, Salkeld et al. 2014a, 2014b) and host trapping (Castro and Wright 2007). However, these studies generally do not link information about ticks and tick-borne diseases directly to human-tick encounters. A more detailed analysis relating the pathogen prevalence in different species of ticks parasitizing humans in the western United States is needed to better assess risk. To address this gap in knowledge, we report results of passive surveillance involving ticks that were submitted from the western United States and tested for pathogens by TickReport at the University of Massachusetts Amherst.

## Materials and Methods

### Ticks and morphological identification

TickReport is a public outreach service at the University of Massachusetts Amherst providing individuals with information about potential pathogen exposures associated with their tick bites and provides information about risk vis-a-vis the biting tick's species, its infection status with respect to several pathogen species, and an assessment of the tick's feeding status. Ticks analyzed in the present study were submitted to TickReport from July 2006 through August 2017. Orders were placed via an online form where submitters are asked to provide the location and date of tick collection; age, gender, and species of the host; and attachment site of the tick on the host's body (Xu et al. 2016).

The *Ixodes* genus-level identification of each tick was determined by morphological characterization of the anal groove, which is distinctively located anterior to the anus in all *Ixodes* ticks (Keirans and Clifford 1978).

### DNA extraction and molecular identification

DNA was extracted from each tick using Epicenter Master Complete DNA and RNA Purification Kits (Epicenter Technologies, Madison, WI) following the manufacturer's protocols. Differentiation of *I. scapularis* and *I. pacificus* was performed by a species-specific TaqMan PCR assay (Table 1). Other *Ixodes* species were identified by amplifying and sequencing a fragment of the tick 16S rRNA gene (Table 1) (Krause et al. 2016, Xu et al. 2016).

**Table 1.** Primers and Probes Used in This Study

*Target gene*	*Application*	*Type*	*Sequences (5′-3′)*	*Tm (C)*	*Reference*
16S	Tick species PCR and confirmation	Forward	TGCTGTAGTATTTTGACTATACAAAGG	55	This article
		Reverse	ATCCTAATCCAACATCGAGGTC		
ITS	*Ixodes scapularis* identification	Forward	TGCGTTTTCTTTGAGCAAATGCACGAG	60	This article
		Reverse	GTACGGGATTTTCCACAAACGGTATCCA		
		Probe	TGCGCTTAACCAGTCCTCCTCCTCCTACGA		
ITS	*Ixodes pacificus* identification	Forward	CTCGGAGCAAGTACGGAGGTAG	60	This article
		Reverse	TTTCCACAAAACGGTCGCCATC		
		Probe	CTGAGCCAAGTCCTCTTCCTACCCGGTTTG		
P13	EMLA detection	Forward	TACCTAATTCTTCTCAAGAGATTCAGTTG	60	This article
		Reverse	ATGATGATACTGCGAACAACTATAAGAG		
		Probe	ATATTGATAAAAGAGTCAGTGTTGATCCGTATGAGTTAGGGTT		
glpQ	*Borrelia miyamotoi* detection	Forward	GACATAGTTCTAACAAAGGACAATATTCC	60	Krause et al. (2016)
		Reverse	TCCGTTTTCTCTAGCTCGATTGG		
		Probe	TGCACGACCCAGAAATTGACACAACCACAA		
ospA	*Borrelia burgdorferi Sensu Lato* detection	Forward	ATAGGTCTAATATTAGCCTTAATAGCAT	60	This article
		Reverse	AGATCGTACTTGCCGTCTT		
		Probe	aagc+Aaa+Atgtt+Agc+Agccttga (LNA probe)		
Tubulin	*Babesia* detection	Forward	GATTTGGAACCTGGCACCATG	60	Xu et al. (2016)
		Reverse	AATGACCCTTAGCCCAATTATTTCC		
		Probe	ATCTGGCCCATACGGTGAATTGTTTCGC		
MSP-2	*Anaplasma* detection	Forward	ATGGAAGGTAGTGTTGGTTATGGTATT	60	Xu et al. (2016)
		Reverse	TTGGTCTTGAAGCGCTCGTA		
		Probe	TGGTGCCAGGGTTGAGCTTGAGATTG		

EMLA, *Ehrlichia muris*-like agent.

*B. burgdorferi s. l.*, *B. miyamotoi*, *B. mayonii*, *B. microti*, *A. phagocytophilum*, and *Ehrlichia muris*-like agent (EMLA) were detected by a multiplex TaqMan PCR assay targeting different genes (Table 1). *Borrelia* detection was performed by first applying a genus-specific detection assay for a conserved target, followed by specific qPCR assays for each of the three species (*B. burgdorferi s. l.*, *B. miyamotoi*, *B. mayonii*). All *B. burgdorferi s. l.* isolates were further differentiated by multilocus sequence analysis (MLSA) using eight housekeeping loci (*clpA*, *clpX*, *nifS*, *pepX*, *pyrG*, *recG*, *rplB*, and *uvrA*) (Margos et al. 2017).

## Results

### Tick species, geographical distributions, and seasonal activity

From July 2006 to August 2017, we received and identified 601 *Ixodes* ticks (58 from 2006 to 2013 and 543 from 2014 to 2017) from California, Oregon, and Washington, of which 549 (91.3%) had bitten humans, 43 were from dogs, 3 were from cats, and 6 were from lawns or unknown sources. The majority of submissions were received from 2014 to 2017. Analyses of ticks and associated pathogens were restricted to the 549 *Ixodes* ticks found on human subjects submitted from these three states (Table 2).

**Table 2.** Pathogen Infection Rates Among *Ixodes* Ticks Submitted to TickReport Testing Service at UMass, Amherst

			*No. of infected ticks/total (%)*						
*Tick species*	*State*	N	Borrelia *genus*	*BBSL*	*BOMI*	*BOMA*	*BAMI*	*ANPH*	*EMLA*
*Ixodes angustus* (*n* = 21)	CA	2	—	—	—	—	—	—	—
	OR	1	—	—	—	—	—	—	—
	WA	18	—	—	—	—	—	—	—
*Ixodes pacificus* (*n* = 381)	CA	302	6/302 (1.99)	4/302 (1.32)	3/302 (0.99)	—	—	2/302 (0.66)	—
	OR	48	2/48 (4.17)	1/48 (2.08)	1/48 (2.08)	—	—	—	—
	WA	31	—	—	—	—	—	—	—
*Ixodes spinipalpis* (*n* = 28)	CA	13	1/13 (7.69)	1/13 (7.69)	—	—	—	2/13 (15.38)	—
	OR	3	—	—	—	—	—	—	—
	WA	12	3/12 (25.00)	3/12 (25.00)	—	—	—	1/12 (8.33)	—
Endemic total		430	12/430 (2.79)	9/430 (2.09)	4/430 (0.93)	—	—	5/430 (1.16)	—
*Ixodes cookei* (*n* = 1)	CA	1	—	—	—	—	—	—	—
*Ixodes holocyclus* (*n* = 1)	CA	1	—	—	—	—	—	—	—
*Ixodes ricinus* (*n* = 6)	CA	6	—	—	—	—	—	1/6 (16.67)	—
*Ixodes scapularis* (*n* = 111)	CA	71	18/71 (25.35)	17/71 (23.94)	1/71 (1.41)	—	1/71 (1.41)	3/71 (4.23)	—
	OR	18	6/18 (33.33)	5/18 (27.78)	1/18 (5.56)	—	1/18 (5.56)	2/18 (11.11)	—
	WA	22	9/22 (40.91)	8/22 (36.36)	1/22 (4.55)	—	2/22 (9.09)	3/22 (13.64)	—
Nonendemic total		119	33/119 (27.73)	30/119 (25.21)	3/119 (2.52)	—	4/119 (3.36)	9/119 (7.56)	—

Tick species was determined unambiguously by DNA sequencing. One *I. pacificus* tick from CA was coinfected with *Borrelia burgdorferi s. l.* and *Borrelia miyamotoi*. For 119 nonendemic ticks, 107 tick bite victims had travel history to Midwest, east coast of the United States, or other countries.

“—”, Indicated no positive ticks were detected for the corresponding pathogen.

ANPH, *Anaplasma phagocytophilum*; BAMI, *Babesia microti*; BBSL, *Borrelia burgdorferi sensu lato*; BOMA, *Borrelia mayonii*; BOMI, *Borrelia miyamotoi*; EMLA, *Ehrlichia muris*-like agent.

Among the 549 human-biting *Ixodes* ticks, three species endemic to the western United States were identified: *I. pacificus* (*N* = 381: 2 larvae, 57 nymphs, 322 adults), *I. spinipalpis* (*N* = 28 nymphs), and *I. angustus* (*N* = 21: 17 nymphs, 4 adults). *I. angustus* and *I. spinipalpis* are considered occasional human biters (Merten and Durden 2000, Eisen, et al. 2006), however, 11.40% (49/430) of the 430 endemic ticks recovered from humans were *I. angustus* (4.88%, 21/430) and *I. spinipalpis* (6.52%, 28/430).

Surprisingly, we also found that 21.67% (119/549) of the ticks from California, Oregon, and Washington residents are not endemic to the western United States. These four tick species are as follows: *I. scapularis* (*N* = 111: 8 larvae, 47 nymphs, 56 adults), *I. ricinus* (*N* = 6: 5 nymphs, 1 adult), *Ixodes cookei* (*N* = 1 nymph), and *Ixodes holocyclus* (*N* = 1 adult). Among the 119 nonendemic ticks submitted, 107 (89.9%) bit people who reported in the TickReport order questionnaire, a travel history to the eastern or north-central United States or foreign countries. No travel history was volunteered for the remaining 12 individuals, but in all likelihood, these nonendemic ticks were also acquired during travel. Based on the travel history, it is clear that the 5 *I. ricinus* ticks and the single *I. holocyclus* tick were brought to the United States from Europe and Australia, respectively.

Over 94.2% of total ticks were submitted from Oregon, Northern California, and Washington. Only a small proportion, 5.8% (*N* = 25), of ticks were submitted from Southern California. For the three endemic tick species: 85.7% of *I. angustus* ticks were submitted from Washington; 79.3% of *I. pacificus* ticks were submitted from California; and 46.4% and 42.9% of *I. spinipalpis* ticks were submitted from California and Washington, respectively (Table 2).

**FIG. 1.**
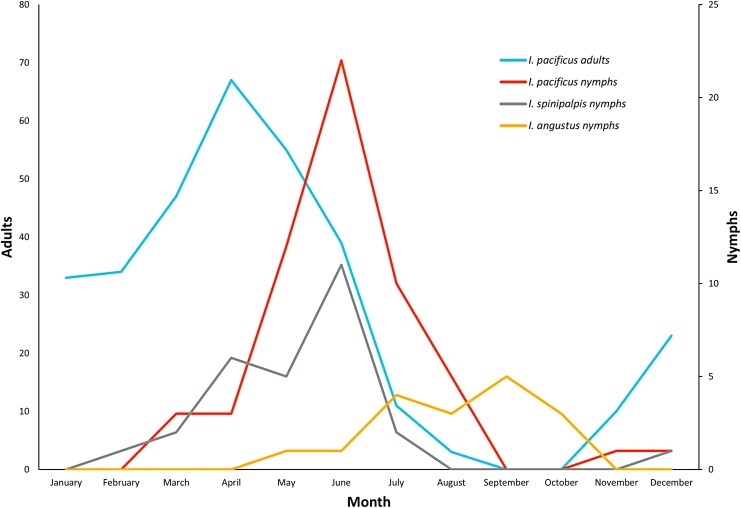
Monthly submission of *Ixodes pacificus* (adults, nymphs), *Ixodes spinipalpis* (nymphs), and *Ixodes angustus* (nymphs) from July 2006 through August 2017.

We received *I. pacificus* in every month of the year except September and October. The nymphal and adult *I. pacificus* exhibited different seasonal activity patterns (Fig. 1). We received 96.5% of nymphs between March and August, with a clear peak in June. Adult *I. pacificus* were abundant from November to July and then absent from September and October, with a peak in April (Fig. 1). We only found nymphal human-biting *I. spinipalpis* in this study. They were submitted between February and July, with a peak in June. Nymphal *I. angustus* were submitted from May to October, with a peak in September (Fig. 1).

### Age distribution and tick attachment sites of tick bite victims

**FIG. 2.**
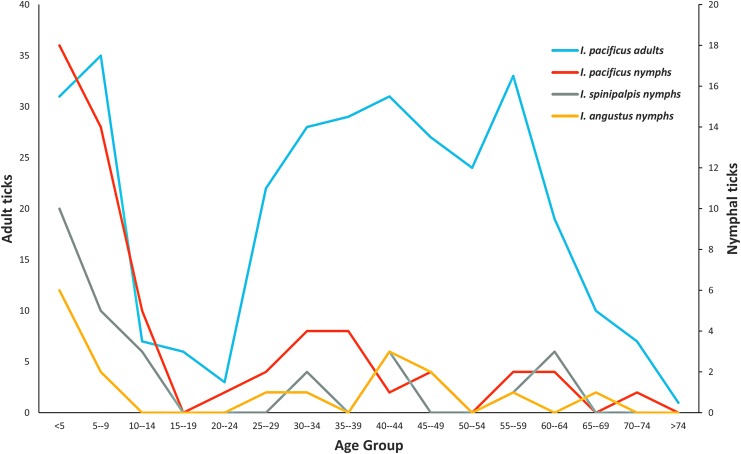
Age distribution of *Ixodes pacificus* (adults, nymphs), *Ixodes spinipalpis* (nymphs), and *Ixodes angustus* (nymphs) bite victims from July 2006 through August 2017.

We received age data for 413 of the tick bite victims (Fig. 2). The age pattern was quite different for humans bit by *I. pacificus* nymphs compared to adults: 22% of attached adults and 57% of attached nymphs were found in children younger than 9 years. Adult ticks (70%) were commonly found among the age group of 25–64 years, however, only 30% of nymphs were found among this age. The individuals aged 0–9 years also had the largest proportion of *I. spinipalpis* and *I. angustus* submitted: 56% of *I. spinipalpis* nymphs and 47% of *I. angustus* nymphs (Fig. 2). The number of *I. angustus* adults was too small to compare age groups.

The percentages of *I. pacificus* adults attached to the abdomen/groin, buttocks, chest, head, lower extremities, neck, shoulder/back, and upper extremities were 4% (A/G), 2% (B), 12% (C), 16% (H), 24% (LE), 4% (N), 24% (S/B), and 13% (UE), respectively. The percentages of *I. pacificus* nymphs attached to the same sites were 4% (A/G), 0% (B), 17% (C), 9% (H), 30% (LE), 11% (N), 6% (S/B), and 23% (UE), respectively. The percentages of *I. spinipalpis* nymphs attached to the same sites were 8% (A/G), 12% (B), 8% (C), 8% (H), 31% (LE), 15% (N), 12% (S/B), and 8% (UE), respectively. The percentages of *I. angustus* nymphs attached to the same sites were 8% (A/G), 0% (B), 8% (C), 23% (H), 38% (LE), 0% (N), 8% (S/B), and 15% (UE), respectively. It seems that all these three tick species prefer to attach to the lower extremities, including thigh, leg, ankle, and foot.

### Prevalence of pathogens and MLSA of *B. burgdorferi*

*B. burgdorferi s. l.*, *B. miyamotoi*, and *A. phagocytophilum* were detected in *I. pacificus* ticks: prevalence was 1.31% (5/381), 1.05% (4/381), and 0.52% (2/381), respectively. *B. burgdorferi s. l.* and *A. phagocytophilum* were detected in *I. spinipalpis* ticks: prevalence was 14.29% (4/28) and 10.71% (3/28), respectively (Table 2). The tick species, life stage, and location of these *B. burgdorferi s. l.* positive ticks can be found in Appendix Table A1. No pathogens were detected in *I. angustus* ticks. *B. mayonii*, *B. microti*, and EMLA were not detected in these three endemic tick species.

*B. burgdorferi s. l.*, *B. miyamotoi*, *B. microti*, and *A. phagocytophilum* were detected in nonendemic *I. scapularis* ticks: prevalence was 27.03% (30/111), 2.70% (3/111), 3.60% (4/111), and 7.21% (8/111), respectively. *A. phagocytophilum* was also detected in the single *I. ricinus* tick. No pathogens were detected in the *I. cookei and I. holocyclus* ticks.

*B. burgdorferi s. l.* prevalence in *I. pacificus* nymphs was higher than in adults (3.5% vs. 0.9%). On the contrary, *B. burgdorferi s. l.* prevalence in *I. scapularis* nymphs was lower than that in adults (25.5% vs. 32.1%). *B. burgdorferi s. l.* prevalence was 14.29% (4/28) in *I. spinipalpis* nymphs, but no *I. spinipalpis* adults were submitted in this study.

**FIG. 3.**
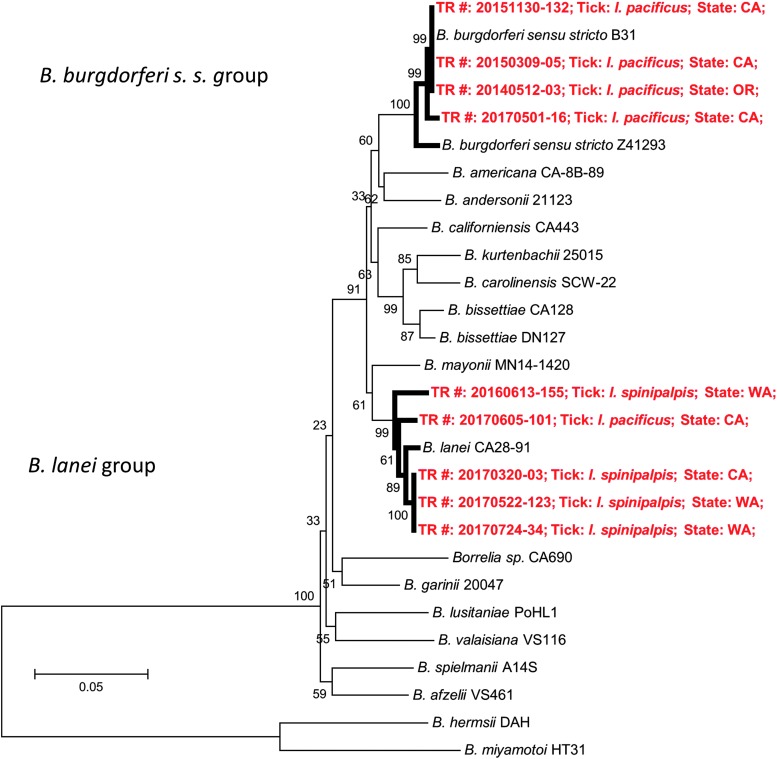
Dendrogram showing only *Borrelia burgdorferi s.s.* and *Borrelia lanei* were detected within *B. burgdorferi sensu lato* in the present study. Reference *Borrelia* strains from GenBank are indicated by species (and strain where applicable). Isolates from the present study are represented by the tick species, in which they were detected (starting with TR#, *red color*). The dendrogram was constructed by minimum evolution method of MEGA 6 software with *Borrelia* relapsing fever group as outgroup. *Numbers* on the branches represent bootstrap support with 1000 bootstrap replicates.

Based upon alignment from MLSA (GenBank accession nos. MH378169–MH378227), we concluded that two members of the *B. burgdorferi s.l.* complex were found in the present study: *B. burgdorferi s. s.* and the recently named *B. lanei* (genomospecies 2) (Schwan et al. 1993, Postic et al. 1994, Margos et al. 2017) (Fig. 3). Of the five *Borrelia*-infected *I. pacificus* ticks, four were infected with *B. burgdorferi s. s.*, and one was infected with *B. lanei*. *B. lanei* was the only *Borrelia* species found in *I. spinipalpis* tick*s*.

## Discussion

The levels of tick infestation, reservoir competence, and *Borrelia* infection prevalence vary widely among hosts in the western United States (Brown and Lane 1992, Eisen et al. 2004, Salkeld et al. 2008, Salkeld and Lane 2010). Passive tick surveillance studies have shown that the nymphal stage of *I. pacificus* is the primary vector of *B. burgdorferi* to humans in California (Clover and Lane 1995). Certain human behaviors, such as sitting on logs, can pose considerable risk of multiple exposures to nymphal ticks in this region (Lane et al. 2004). So too, travel history to the northeastern or upper midwestern United States impacts Lyme disease reporting from California patients diagnosed by an erythema migrans rash. Onset of the rash in patients exposed in California peaked in May–July, while the onset in patients (*N* = 219) with a travel history peaked in June–August (*N* = 177); reflecting differences in tick seasonal activity in each region (Salkeld et al. 2014a). Some tick species, such as *I. spinipalpis* and *I. angustus* are considered “rare” human biters (Damrow et al. 1989, Brown and Lane 1992, Merten and Durden 2000, Peavey et al. 2000), however, their encounter frequency and public health impact are yet to be determined.

In this study, we found that 21.67% of *Ixodes* ticks from California, Oregon, and Washington residents are not endemic to the western United States. These data indicate that tick species identification and travel history are critical factors for human-based tick surveillance in the western United States. Although *I. pacificus* and *I. scapularis* are closely related species, the acarologic risk of exposure to pathogens in these ticks is very different (Eisen et al. 2004, Hahn et al. 2018). Accuracy of species identification is challenging for several reasons. First, *I. pacificus* is very similar to *I. scapularis* morphologically. Second, most human-biting ticks are in poor condition, often with missing mouth parts, further complicating proper identification. Finally, the geographic distributions of these two species do not overlap (Eisen et al. 2016). Therefore, it is often assumed that a human-biting tick in a certain area is representative of the local tick species. In this study, we used real-time PCR and DNA sequencing to overcome these complications and accurately identify each sample to the species level. With proper species identification, we were able to conclude that nonendemic *Ixodes* ticks may influence local tick-borne disease due to human travel between nonoverlapping tick ranges.

Our data indicate that West Coast residents traveling to the northeast United States have a much higher risk of being bitten by an infected tick compared to nontraveling residents who are exposed to local tick populations. We found that 20.22% (111/549) of the total human-tick encounters were cases of *I. scapularis* ticks biting travelers. Second, *B. burgdorferi s. l.*, *B. miyamotoi*, and *A. phagocytophilum* prevalence in *I. scapularis* is 20.6, 2.6, and 13.7 times higher, respectively, compared to *I. pacificus* (Table 2). We do not know how many tick-borne diseases were acquired from traveling for the West Coast residents. However, the 10-year data for humans with erythema migrans in California (2001–2011) showed two different frequency peaks: one is likely local exposure in May–July and another peak is likely travel-associated exposure in June–August (Salkeld et al. 2014). Assessing risk for tick-borne diseases in the western United States without accurate species identification and travel information would likely overestimate the number of local cases.

*I. angustus* and *I. spinipalpis* are normally considered nonhuman feeders but were experimentally demonstrated to be competent vectors of *B. burgdorferi* (Dolan et al. 1997, Merten and Durden 2000, Peavey et al. 2000). In this study, 11.40% (49/430) of the endemic ticks recovered from humans in the western United States were *I. angustus* and *I. spinipalpis*. We found 4.88% (21/430) of human-biting ticks were *I. angustus.* Both nymphal and adult *I. angustus* occasionally quest openly in moist and cool habitats, especially in coastal areas (Eisen et al. 2006). In this study, most *I. angustus* (14/21) were nymphs from Washington.

*I. spinipalpis* is more important than the human-biting “bridge” vector, *I. pacificus*, in maintaining the enzootic spirochete cycle in the western United States (Brown and Lane 1992, Oliver et al. 2003, Eisen et al. 2009). However, we found the human risk for exposure to *I. spinipalpis* and the pathogens it transmits may be underestimated. First, 6.52% (28/430) of the endemic ticks recovered from humans in the western United States were *I. spinipalpis* in this study. This species may use a primarily nidicolous host-seeking strategy in hot and dry climates, but commonly quest for hosts openly in moister habitats (Eisen et al. 2006). In some redwood-associated woodland sites in California, a larger number of openly host-seeking *I. spinipalpis* nymphs were collected by the dragging flag method, compared to *I. pacificus* (Eisen et al. 2006). All human-biting *I. spinipalpis* were nymphal ticks in this study. Second, the pathogen infection rate in *I. spinipalpis* was higher than that in *I. pacificus*: 14.29% (4/28) versus 1.31% (5/381) and 10.71% (3/28) versus 0.52% (2/381) for *B. burgdorferi s. l.* and *A. phagocytophilum*, respectively.

Although only *B. burgdorferi s. s.* and *B. mayonii* in *B. burgdorferi s. l.* complex have been culture confirmed as human pathogens in the United States (Steere et al. 1983, Pritt et al. 2016), several other species, including *B. americana*, *B. bissettiae*, *B. burgdorferi s. s.*, *Borrelia californiensis*, and *B. lanei*, have been found in *I. pacificus* or *I. spinipalpis* ticks (Postic et al. 2007, Rudenko et al. 2009, Fedorova et al. 2014, Margos et al. 2017). Based upon results of MLSA in the present study, human-biting *I. pacificus* and *I. spinipalpis* are associated with only two species within the *B. burgdorferi s. l.* complex: *B. burgdorferi s. s.* and the recently named *B. lanei* (genomospecies 2) (Schwan et al. 1993, Postic et al. 1994, Margos et al. 2017) (Fig. 3). Although the *Borrelia* prevalence was 14.29% (4/28) in *I. spinipalpis* tick*s*, *B. lanei* was the only *Borrelia* species found in this tick species. *B. lanei* was recently found in *I. spinipalpis* ticks collected from rabbits in California and Canada, suggesting that rabbits may play an important role in the transmission cycle of *B. lanei* (Margos et al. 2017, Scott et al. 2017). It is well known that *B. burgdorferi s. s.* is pathogenic to humans in North America; however, human cases of *B. lanei* infection have not been found in the western United States (Margos et al. 2017). The vector role of *I. pacificus* and *I. spinipalpis* in the enzootic cycle of *B. lanei* and the pathogenicity of *B. lanei* to humans warrants further study.

The role of adult *I. pacificus* in disease transmission is not clear, however, we found adult ticks carry *B. burgdorferi*, *B. miyamotoi*, and *A. phagocytophilum* in California and Oregon. The nymphal stage of *I. pacificus* is the primary vector of Lyme disease in California (Clover and Lane 1995), but nymphs are difficult to detect and remove before repletion due to the tick's small body size (Xu et al. 2016). We found that a high rate (57%) of nymph bite-related encounters was in children aged 9 years and younger. Of these nymphal attacks, 96.5% of them were found between March and August, with a clear peak in June. We also received 56% of *I. spinipalpis* nymphs and 47% of *I. angustus* nymphs in victims aged 9 years and younger. In addition, we found that 48% of *I. pacificus* adults were attached to the lower extremities and back, whereas 30%, 31%, and 38% of *I. pacificus*, *I. spinipalpis*, and *I. angustus* nymphs were attached to the lower extremities. To help prevent tick-borne disease, it is critical to educate parents of young children to check for nymphal ticks during March–August in the West Coast, paying close attention to the lower extremities of young children.

## Conclusions

We show that many nonendemic *Ixodes* ticks (119/549) submitted to the TickReport public testing program from the West Coast are most likely acquired from travel to a different geographic region. Traveling to an area where the tick population has a relatively higher pathogen infection rate increases the risk of exposure to infected ticks: as in the case of West Coast residents traveling to the northeast United States. For tick species that are endemic to the West Coast, we report cases of conventionally recognized nonhuman feeders (*I. spinipalpis* and *I. angustus*) parasitizing humans. Importantly, *I. spinipalpis* had the highest pathogen prevalence of the endemic species, indicating that it may pose a larger public health threat than previously thought. Furthermore, two species within the *B. burgdorferi s. l.* complex were detected in West Coast ticks; *B. burgdorferi s. s.* and *B. lanei. B. lanei* was detected in *I. spinipalpis* and *I. pacificus* ticks, corroborating previous reports. The vector role of *I. pacificus* and *I. spinipalpis* in the enzootic cycle of *B. lanei* and the pathogenicity of *B. lanei* to humans warrants further study. Seasonal activity, age distribution, and site of attachment data have yielded valuable public health information: for instance, young children are at an increased risk of tick bites, likely around their lower extremities, especially during the month of June when seasonal activity peaks for both nymphal *I. pacificus* and *I. spinipalpis* ticks. Public health professionals and physicians are encouraged to use this information to limit tick bites and tick-borne pathogens.

These results highlight the importance of passive surveillance of human-biting ticks and pathogens. Epidemiological case reports and/or entomological infection rate of questing ticks provide only proxies of human risk of tick-borne disease. Testing human-biting ticks allows assessment of the biological correlates of risk (tick species, degree of engorgement, and infection status) and the most reliable geographic distribution of that risk.

## Appendix

**Appendix Table A1.** *Borrelia* Positive Samples from Human-Biting Endemic *Ixodes* Ticks in This Study

*TR ID no.*	*Tick species*	*Tick stage*	*Location*	*Tick removed date*	Borrelia *species*
20140512-03	*Ixodes pacificus*	Adult female	Applegate, OR	May 5, 2014	*Borrelia burgdorferi sensu stricto*
20150309-05	*I. pacificus*	Adult female	Santa Rosa, CA	March, 8, 2015	*B. burgdorferi sensu stricto*
20151130-132	*I. pacificus*	Adult female	Santa Cruz Mountains, CA	November 29, 2015	*B. burgdorferi sensu stricto*
20170501-16	*I. pacificus*	Nymph	Glen Ellen, CA	April 24, 2017	*B. burgdorferi sensu stricto* and *Borrelia miyamotoi*
20170605-101	*I. pacificus*	Nymph	San Lorenzo, CA	May 29, 2017	*Borrelia lanei*
20160613-155	*Ixodes spinipalpis*	Nymph	Gig Harbor, WA	June 11, 2016	*B. lanei*
20170320-03	*I. spinipalpis*	Nymph	Half Moon Bay, CA	March 12, 2017	*B. lanei*
20170522-123	*I. spinipalpis*	Nymph	Gig Harbor, WA	May 4, 2017	*B. lanei*
20170724-34	*I. spinipalpis*	Nymph	Gig Harbor, WA	July 15, 2017	*B. lanei*
20150202-03	*I. pacificus*	Adult female	Stanislaus County, CA	January 25, 2015	*Borrelia miyamotoi*
20170327-22	*I. pacificus*	Adult female	Salinas, CA	March 26, 2017	*B. miyamotoi*
20170403-97	*I. pacificus*	Adult female	Rogue River, OR	March 30, 2017	*B. miyamotoi*

The GenBank accession numbers of deposited MLSA sequences are MH378169–MH378227.

MLSA, multilocus sequence analysis.
